# Human Lingual Biofilm Signatures in Gastrointestinal Disease: A Scoping Review

**DOI:** 10.1016/j.identj.2026.109762

**Published:** 2026-07-28

**Authors:** Kausar Sadia Fakhruddin, Moyad Shahwan, Ayaana Kamal, Osman Elmobarik, Thitaya Chaiboonyarak, Alexander Patera Nugraha, Han-Sung Jung, Lakshman Samaranayake, Thantrira Porntaveetus

**Affiliations:** a Faculty of Dentistry, The University of Western Australia, Nedlands, Western Australia, Australia; b Faculty of Dentistry, University of Saskatchewan, Saskatchewan, Canada; c Center of Excellence in Precision Medicine and Digital Health, Geriatric Dentistry and Special Patients Care International Program, Department of Physiology, Faculty of Dentistry, Chulalongkorn University, Bangkok, Thailand; d Center of Medical and Bio-Allied Health Sciences Research at the College of Pharmacy and Health Sciences, Ajman University, Ajman City, United Arab Emirates; e Faculty of Medicine, Georgian National University SEU, Tbilisi, Georgia; f Dental Regenerative Research Group, Department of Orthodontic, Faculty of Dental Medicine, Universitas Airlangga, Surabaya, East Java, Indonesia; g Division in Anatomy and Developmental Biology, Department of Oral Biology, Taste Research Center, Oral Science Research Centre, BK21 FOUR Project, Yonsei University College of Dentistry, Seoul, South Korea; h Dr DY Patil Dental College and Hospital, Dr DY Patil Vidyapeeth, Pune, India; i Faculty of Dentistry, University of Hong Kong, Hong Kong, China; j Clinic of General, Special Care and Geriatric Dentistry, Centre for Dental Medicine, University of Zurich, Zurich, Switzerland

**Keywords:** Tongue microbiome, Gastrointestinal diseases, Precancerous lesions, Biomarkers, Oral-gut axis, Healthcare access

## Abstract

**Introduction and aims:**

The tongue dorsum represents a structurally complex oral biofilm niche that has traditionally been regarded as indicative of systemic health. Recent advances in oral microbiome research and multi-omics technologies facilitate the systematic evaluation of the association between tongue coating biofilm signals and gastrointestinal disease states. However, it remains unclear whether these tongue-derived signals indicate systemic gastrointestinal pathology or merely reflect localised oral ecological disturbances. This review synthesises current evidence on tongue-derived microbial and multi-omics signatures across inflammatory, precancerous, and malignant gastrointestinal conditions, and evaluates their ecological, biological, and clinical significance.

**Methods:**

A scoping review was conducted in accordance with PRISMA-ScR guidelines. Five electronic databases were searched (2010-2025) for human studies analysing tongue-coating samples using microbiome or multi-omics approaches.

**Results:**

A total of twenty-five cross-sectional studies involving more than 4500 participants, primarily from East Asian populations, were included. Three recurrent patterns were identified: (1) stage-associated microbial restructuring, which involved mild non-specific alterations in inflammatory states, structured dysbiosis in precancerous conditions, and more consistent ecological configurations in malignancy; (2) convergence of functional multi-omics signals on lipid metabolism pathways across independent cohorts; and (3) significant modification of microbial and functional profiles by tongue coating phenotype, including colour, thickness and classification system.

**Conclusions:**

Tongue-derived microbial and multi-omics signatures demonstrate reproducible cross-sectional associations with gastrointestinal diseases, exhibiting functional convergence across multiple omics layers. However, the reliance on cross-sectional study designs, absence of external validation and insufficient adjustment for confounding variables currently limit their clinical application as diagnostic biomarkers.

**Clinical relevance:**

Tongue examination is clinically useful for assessing oral biofilm burden, mucosal pathology and oral hygiene in dental practice. It should not be used to diagnose gastrointestinal disease until validated by longitudinal, confounder-controlled studies.

## Introduction

The dorsal surface of tongue constitutes a distinct ecological niche defined by papillary microarchitecture that promotes microbial retention and biofilm development. Historically, this anatomical region has been considered a diagnostic indicator of internal health for more than two millennia, especially within traditional Chinese medicine, where features such as tongue coating characteristics, colour, thickness and moisture are interpreted as reflections of organ system status.

Contemporary dentistry recognises the tongue dorsum as a structurally complex and spatially organised environment composed of desquamated epithelial cells, diverse microbial populations and their metabolites, and host proteins, collectively forming a persistent biofilm. Unlike self-cleansing non-keratinised oral mucosal surfaces, the tongue coating accumulates biological material, rendering it a valuable sampling site for the study of oral-systemic interactions.^1^^,^^2^ However, it remained unclear whether signals from this niche solely reflect localised oral ecological variations also provide meaningful insights into gastrointestinal (GI) disease processes.

In the past two decades, the concept of the 'oral-gut axis' has provided a framework for understanding the connections between oral and gastrointestinal (GI) microbial ecosystems. The act of swallowing introduces oral microorganisms into the GI tract, resulting in ongoing microbial exchange between these environments.^3^^,^^4^ The presence of oral taxa in gastric and intestinal sites, including in GI malignancies, suggests a potential association between oral microbial communities and GI disease processes. However, it remains unclear whether these findings reflect biologically significant translocation or parallel ecological changes driven by shared exposures (such as diet, medications and inflammatory events), host physiology, environmental factors, or clinical interventions.^5^, ^6^, ^7^ Furthermore, the influence of proton pump inhibitor (PPI) use, oral hygiene practices, dietary patterns and lifestyle factors on tongue biofilm composition has not been sufficiently investigated.

Recent advances in high-throughput sequencing and multi-omics technologies have facilitated comprehensive characterisation of tongue-derived signals beyond taxonomic profiling. Nevertheless, this technological progress has resulted in considerable heterogeneity in study design, analytical platforms and outcome measures. Furthermore, the predominance of cross-sectional study designs inherently restricts the ability to draw causal inferences.

This scoping review pursues three primary objectives. First, to synthesise and critically interpret tongue-derived microbial and multi-omics signatures across inflammatory, precancerous and malignant gastrointestinal conditions, focusing on cross-sectional evidence. Second, to evaluate the convergence of multi-omics signals, with particular emphasis on metabolic pathways. Third, to assess the extent to which these signals are influenced by tongue coating phenotypes, including colour, thickness and classification systems. Collectively, the review seeks to differentiate exploratory biological associations from clinically actionable biomarkers and to delineate the methodological requirements for translation into screening, risk stratification, or diagnostic application.

## Material and methods

### Study design

The scoping review was conducted in accordance with PRISMA-ScR guidelines as detailed in Table S1^8^ and was registered with Open Science Framework (OSF): https://doi.org/10.17605/OSF.IO/BW8QV, https://osf.io/bw8qv/metadata/osf

### Search strategy

Five databases (PubMed/MEDLINE, Scopus, Embase, EBSCOhost, Web of Science) were searched from 1st January 2010 to 31st December 2025. A final search update was performed in February 2026. Search strategies combined terms for: (1) tongue/coating (e.g., ‘tongue microbiome‘); (2) multi-omics profiling (‘microbiota‘, ‘metabolomics‘, ‘proteomics‘); and (3) gastrointestinal disease (‘gastritis‘, ‘precancerous lesions‘, ‘dysplasia‘, ‘gastrointestinal cancer‘). Full search strategies are in Supplementary Text S1.

### Eligibility criteria

*Inclusion*: Human studies of inflammatory, precancerous, or malignant GI conditions; tongue-derived samples (swabs/scrapings); microbial or multi-omics outcomes; observational designs; English; 2010-2025. *Exclusion*: Saliva/plaque/buccal samples without tongue data; animal/*in vitro* studies; reviews, abstracts, or case reports; insufficient methodology; tongue appearance without molecular characterisation.

### Study selection and appraisal

Both data charting and the JBI methodological appraisal were performed initially by a single reviewer (KSF) and independently verified by a second reviewer (AK). Any disagreements were resolved through discussion and mutual consensus. Duplicates were removed, after which a two-stage screening process (title and abstract, followed by full text) discrepancies were resolved through consensus. The selection is summarised in Figure S1. Data were extracted for author, year, country, study design, sample size, disease, sampling method, platform, confounders and key findings (Table 1, Table 2, Table 3). Methodological quality was assessed using the Joanna Briggs Institute domains,^9^ without formal scoring due to the scoping nature of the review (Tables S2-S4). Confidence levels in Table 4 were assigned qualitatively based on four domains: study design, sample size, confounder adjustment and external validation. Low: Cross-sectional studies with small sample sizes and limited confounder control; Moderate: Multiple studies showing consistent findings but lacking external validation; Higher: Reproducible findings across larger cohorts with partial confounder adjustment.

**Table 1:** Tongue-derived microbial signatures in inflammatory and precancerous gastrointestinal conditions.

Study (Year)Country	Pre-cancer typeDesign; N = (case/control)	Tongue samplingplatform	Confounders addressedmissing confounders	Effect direction summary
Yanan et al.^10^ China	CAG (chronic atrophic gastritis, a precancerous condition for gastric cancer) vs CSG (chronic superficial gastritis). Case-control; N = 158 chronic gastritis (CG)/30 healthy controls (HC)	Tongue scraper (disposable sterile swab, 5 strokes, middle dorsum) 16S rDNA (V3-V4), Illumina NovaSeq	Age, sex (frequency-matched) Diet, smoking, drinking, PPI/antibiotics (excluded but not adjusted) and oral hygiene.	CG vs HC: ↑ alpha diversity (Chao1, Shannon) in CG. Phylum: Bacteroidetes ↓ (37.50% vs 40.41%), Proteobacteria ↑ (26.54% vs 24.83%), Fusobacteria ↑ (11.53% vs 7.84%). Genus: *Neisseria, Prevotella_7, Haemophilus, Porphyromonas, Fusobacterium* changed. Four tongue fur types (WN/WK/YN/YK) showed significant differences in microbial composition. YK (thick yellow) had the highest diversity.
Xiao et al.^11^ China	Gastric precancerous lesions- intestinal metaplasia, dysplasia Case-control; N = 60 GPL/20 healthy controls	Sterile swab thickest tongue coating area, 5 strokes 16S rDNA (V3-V4), Illumina Hiseq 2500 (2 × 250 paired ends)	Age, sex, *H. pylori* status Diet, smoking, alcohol (excluded by criteria), BMI (excluded if >28), oral hygiene, probiotics/antibiotics use (excluded if taken), PPI use	GPL vs. HC: ↑ *Solobacterium, Rothia, Oribacterium, Alloprevotella* Damp phlegm vs. non-damp phlegm GPL: ↓ *Terrisporobacter, Solobacterium, Porphyromonas, Parvimonas, Lactobacillus, Johnsonella, Gemella, Fusibacter, Azoarcus, Acidothermus*
Xiao et al.^12^ China	Oesophageal precancerous lesions Case-control; N = 123 EPL pts./76 healthy controls	Tongue scraper (sterile toothbrush, middle dorsum) 16S rDNA (V3-V4), Illumina MiSeq	Age, sex, BMI (matched); Hp infection status Diet details, education, income, oral hygiene, antibiotics/PPI use (excluded but not adjusted)	EPL vs. HC: ↑ *Peptostreptococcus, Capnocytophaga*, [*Eubacterium*] yuri group; ↓ *Atopobium, Hydrobacter, Taonella*. No alpha diversity difference. Weakened symbiotic network.
Kang et al.^13^ China	Precancerous lesions of the upper gastrointestinal tract (PLUGT): gastric (PLGC) and oesophageal (ESCC) Case-control; 153 PLUGT (47 W-thin, 19 W-thick, 47 Y-thin, 40 Y- thick)/ 47 controls (white-thin, healthy)	Tongue coating is used separately for microbiome (16S rRNA). Peripheral blood serum (cytokines/TBA) ELISA measured 1 cytokine (IL-17A) and TBA. (Human IL-17A ELISA KIT, Total Bile Acid ELISA Kit)	Age, sex Diet, smoking, alcohol, oral hygiene, probiotics/antibiotics (excluded if taken within 4 weeks), periodontitis/oral disease (excluded), PPI use not mentioned	*Parvimonas* was positively correlated with IL-17A in W-thick and Y-thin coatings; *Dialister* was positively correlated with TBA in W-thin and Y-thick coatings; *Streptococcus* (oral dominant) was negatively correlated with TBA in the Y-thin coating. Serum IL-17A: significantly higher in W-thin group vs. controls (*P* < .05); TBA: no significant difference between any PLUGT group and controls.
Hao et al.^14^ China	Gastric precancerous lesions (GPL) – intestinal metaplasia + dysplasia Case-control; N = 60 GPL/15 healthy controls	Sterile swab, thick part of tongue dorsum, 5 strokes, into sterile tube Microbiome: 16S rRNA (V3-V4), Illumina Hiseq 2500 Metabolomics: GC-TOF-MS + UHPLC-QE-MS	Age, *H. pylori* Diet, smoking, alcohol (excluded), BMI (excluded if >28), oral hygiene, probiotics/antibiotics (excluded), PPI use not mentioned	GPL vs. HC: ↑ *Alloprevotella, Solobacterium, Rothia, Eikenella, Aggregatibacter*; ↓ one uncultured bacterium.
Cui et al.^15^ China	Gastritis (superficial gastritis, atrophic gastritis, intestinal metaplasia) – precancerous cascade for gastric cancer Case-control; N = 78 gastritis (44 SG, 11 AG, 23 IM)/50 healthy controls	Tongue-coating swab, root to tip 30 times while rolling, into PBS, repeated twice Metagenomic shotgun sequencing (not 16S); Illumina HiSeq 2000 / HiSeq 2500	Age, sex, *H. pylori* status, spicy food preference Diet (other than spicy preference), smoking, alcohol, oral hygiene, probiotics/antibiotics (excluded if <3 months), PPI use not mentioned	Gastritis vs. HC: ↑ *Streptococcus infantis, Treponema vincentii, Leptotrichia unclassified, Campylobacter rectus, Campylobacter showae, Capnocytophaga gingivalis, Leptotrichia buccalis, Campylobacter concisus, Selenomonas flueggei, Leptotrichia hofstadii*; ↓ *Veillonella parvula, Corynebacterium matruchotii, Kingella oralis, Atopobium rimae, Aggregatibacter aphrophilus, Streptococcus sanguinis, Acinetobacter lwoffii, Prevotella amnii, Prevotella bivia, Cardiobacterium hominis, Oribacterium sinus* Precancerous cascade (SG → AG → IM): ↑ *Campylobacter concisus* abundance (*P* = .02 for trend; *P* = .004 for first 3 stages: healthy, SG, AG)

HC, Healthy controls; CG, Chronic gastritis; CAG, Chronic atrophic gastritis; SG, Superficial gastritis; AG, Atrophic gastritis; IM, Intestinal metaplasia; GPL, Gastric precancerous lesions; EPL, Oesophageal precancerous lesions; PLUGT, Precancerous lesions of the upper gastrointestinal tract; TBA, Total bile acids; PPI, Proton pump inhibitor.

This table summarises studies examining alterations in the tongue-derived microbiome in inflammatory gastrointestinal conditions and histologically confirmed precancerous lesions, including gastritis, intestinal metaplasia, dysplasia and oesophageal precancer. Findings are presented in relation to microbial composition, changes in diversity and phenotype-dependent stratification.

Key Pattern Identified: Across inflammatory and precancerous gastrointestinal conditions, tongue-derived microbial profiles demonstrate a transition from mild, non-specific alterations in inflammatory states to more structured dysbiosis in precancerous lesions. This is characterised by enrichment of anaerobic taxa and loss of commensal organisms, with consistent evidence that phenotype-associated stratification (e.g., coating colour and thickness) modifies microbial composition and network behaviour.

**Table 2:** Tongue-derived microbial signatures in gastrointestinal and related malignancies: study characteristics, methodology and directional microbiome changes.

StudyCountry	Cancer typeDesign; N=(case/control)	Tongue sampling areaPlatform	Confounders addressedMissing confounders	Effect direction summary (control or comparator)
Liu et al.^16^ China	Colorectal Cancer Cross-sectional (3 groups: CRA, CRC, HC) N = 377 total (65 CRA, 256 CRC / 56 HC)	Tongue coating was scraped at 360 degrees across different tongue areas 16S rRNA (V3–V4); Illumina MiSeq PE250	Age, gender, BMI, antibiotic use (excluded if within 3 months), smoking, drinking, diabetes, obesity Diet (detailed), PPI use, oral hygiene (beyond rinsing), periodontal status	Tongue features: Thick, greasy and peeling coating increased from Healthy → CRA → CRC (*P* < .05). Microbiome: *Prevotella* decreased in CRC; *Actinomyces* and *Leptotrichia* increased progressively. Core species in CRC: *Porphyromonas, Actinomyces, Alloprevotella, Oribacterium, Haemophilus*
Ma et al.^17^ China	Digestive system tumours (including GC, CRC, EC, HPB) Cross-sectional comparative study, N = 1199 (710 DST / 489 HC)	Swab scraping from the posterior → anterior central dorsum of the tongue 16S rRNA (V3–V4; NovaSeq (2 × 250 bp)	Age, sex, smoking, drinking (via PSM) Diet (not explicitly via PSM in methods), PPI use (not mentioned)	DST vs. HC: ↑ *Alloprevotella, Prevotella, Campylobacter, Fusobacterium (GC, CRC);↓* *Neisseria, Haemophilus, Porphyromonas*
Wang et al.^18^ China	Oesophageal adenocarcinoma Case-control, N = 56 (28 EAC /28 HC)	Swab scraping from the middle of the tongue 16S rRNA (V3-V4, Illumina MiSeq PE300)	Age, sex, smoking, drinking, antibiotic use (excluded if within 6 months) Diet (detailed), oral hygiene (beyond gargling), BMI, periodontal status	EAC vs. HC: ↑ *Leptotrichia, Porphyromonas, Peptostreptococcus, Tannerella, Johnsonella; ↓* ↓ *Streptococcus, Rothia*; Increased Shannon diversity in EAC (*P* = .033)
Zheng et al.^19^ China	Colorectal cancer Cross-sectional/descriptive, N = 59 (CRC patients only; no healthy controls)	Swab scraping from the front and middle tongue regions 16S rRNA full-length sequencing (PacBio, V1-V9)	Age, gender, disease stage, location, tongue image type, antibiotic use (excluded if within 1 month) BMI, detailed diet (beyond "comparable habits"), PPI use, smoking, drinking, oral hygiene (beyond "gargle with water")	Yellow greasy coating: associated with *Alloprevotella rava*; White greasy coating: associated with *Prevotella intermedia; Petechiae*: associated with *Neisseria cinerea* and *N. flavescens_A*; Age <62 years: associated with *Streptococcus parasanguinis*; Age ≥62 years: associated with *Prevotella* sp.
Chen et al.^20^ China	Colorectal cancer + colorectal polyps Case-control; N = 90 (30 CRC/30 CP/30 HC)	Swab scraping posterior→anterior middle dorsum of the tongue Metagenomic shotgun sequencing (MGI DNBSEQ-T7, 150 bp PE, ∼7.72 Gb/sample)	Age, gender, BMI, smoking, drinking Diet, PPI use and oral hygiene status, tumour stage not fully integrated into microbiome model	Enriched in CRC: *Actinobacteria (phylum), Atopobium rimae, Veillonella atypica, Megasphaera micronuciformis, Prevotella spp.* Depleted in the CRC: Proteobacteria*, Neisseria, Haemophilus*
Nie et al.^21^ China	Tumour tissue, paracancerous tissue, cancer surface swab, contralateral normal mucosa swab, saliva, tongue coat Case-control; N = 65 OSCC patients (no healthy controls; 305 samples from 6 niches, within OSCC patients, comparing niches)	‘Tongue coat’ niche using a sterile swab 16S rDNA (V3-V4), Illumina HiSeq 2500	Age, sex, cancer site, tumour size, grade, stage, metastasis (no antibiotics in past 3 months, no other systemic diseases) Diet (detailed); PPI use; Oral hygiene status (beyond pre-sampling instructions); Periodontal status; BMI; Tea/coffee consumption; HPV status; Betel nut chewing; Multivariable adjustment for tumour stage/site (only used for stratification, not as a confounder in main models)	Enriched in tumour tissue vs. paracancerous: *Fusobacterium, Prevotella, Porphyromonas, Campylobacter, Aggregatibacter, Treponema, Peptostreptococcus;* Depleted: *Stenotrophomonas, Neisseria, Sphingomonas, Veillonella;* Metastasis-associated genera: *Lautropia, Asteroleplasma, Parvimonas, Peptostreptococcus, Pyramidobacter, Roseburia, Propionibacterium*
Zhang et al.^22^ China	Primary liver cancer, including Hepatocellular carcinoma, intrahepatic cholangiocarcinoma, mixed hepatocellular cholangiocarcinoma Case-control; N = 85 (60 PLC: 30 LTG, 30 LNTG/25 HC)	Fresh one-off toothbrush, middle section of the tongue dorsum 16S rRNA (V3-V4, Illumina MiSeq)	Age, sex, BMI, diabetes, high blood pressure, antibiotic use (excluded if within 2 months) Diet (detailed), oral hygiene (beyond rinsing), PPI use, periodontal status	PLC vs. HC: *Firmicutes* and *Actinobacteria* ↑ in PLC; *Fusobacteria, Spirochaetes* ↓ in PLC. Thick/greasy coating (LTG) vs. non-thick/greasy (LNTG): *Actinobacteria, Veillonella, Actinomyces, Prevotella_7* ↑ in LTG. A 15-genus signature distinguished PLC from HC Key discriminatory genera: *Fusobacterium, Bergeyella, Megasphaera, Prevotella_6, Veillonella, Peptococcus, Solobacterium*
Chung et al.^23^ USA	Pancreatic cancer and periampullary adenocarcinoma, extrahepatic cholangiocarcinoma and other pancreatic conditions Cross-sectional (within-subject, multiple sites) N = 52 subjects with GI cancer	Tongue swab (sterile cytology brush) Illumina (paired-end, V3-V4 region)	Age, sex, race, BMI, smoking, chemotherapy, antibiotic use (within-subject correlation, disease status via ICD-10) Diet, PPI use, oral hygiene, clinical periodontal status	Shared ASVs between oral and pancreatic/intestinal sites: *Streptococcus, Veillonella, Prevotella, Fusobacterium, Gemella, Haemophilus, Rothia*; Significant concordance (Kappa): *Fusobacterium nucleatum subsp. vincentii, Rothia mucilaginosa, Gemella morbillorum, Rothia aeria;* PASTA-identified: *Gemella morbillorum, Fusobacterium nucleatum subsp. vincentii*
Xu et al.^24^ China	Gastric cancer (adenocarcinoma) Case-control N = 181 GC, 112 HCs (original); 66 GC, 66 HCs after PSM	Tongue scraper; posterior to anterior middle area of tongue dorsum Illumina MiSeq (16S V3-V4 + 18S ITS1-2) Serum (peripheral blood) UPLC-Q-TOF/MS	Age, gender, smoking, drinking, diet (via PSM) PPI, antibiotics, oral hygiene	Enriched in GC vs. HCs: *Lactococcus, Megamonas, Granulicatella, Geobacillus, Pseudomonas, Lactobacillus, Carnobacterium, Bacteroides, Faecalibacterium, Escherichia-Shigella, Lachnoclostridium, Leuconostoc;* Depleted: *Porphyromonas, Parvimonas, Capnocytophaga, Peptostreptococcus, Peptococcus, Rothia, Neisseria* Significantly increased in GC: IL-17α, IL-12/IL-23P40, IFN-γ, IL-10 Significantly decreased in GC: IL-1α, IL-5, TNF-β, VEGF IL-17α was negatively correlated with HC-enriched genera: *Porphyromonas, Capnocytophaga, Parvimonas* (r < -0.35).
Lu et al.^25^ China	Pancreatic head carcinoma Case-control; N = 30/25 (PHC / healthy controls) + comparison to 35 LC patients	Tongue scraper, posterior to anterior middle area 16S rRNA (V3-V4), Illumina MiSeq	Age, gender, BMI, oral hygiene (brushing twice daily), antibiotics (excl. 3 months) Smoking, drinking, diet, PPI use, tea consumption, tumour stage (only stage I included), lifestyle details	Enriched in PHC: *Leptotrichia, Fusobacterium, Rothia, Actinomyces, Corynebacterium, Atopobium, Peptostreptococcus, Catonella, Oribacterium, Filifactor, Campylobacter, Moraxella, Tannerella* Depleted in PHC: *Haemophilus, Porphyromonas, Paraprevotella*
Xu et al.^26^ China	Gastric cancer Case-control; N = 115/35 (GC patients / healthy controls -white-thin coating)	A sterile toothbrush from the middle section of the tongue dorsum 16S rRNA (V3-V5) + 18S rRNA (ITS1-2)	Age, gender Lifestyle (smoking, drinking, tea), pathological type, clinical treatment, PPI use, antibiotics use (exclusion criteria: within past 4 weeks), oral hygiene	White-thin (W-thin): *↑ Capnocytophaga leadbetteri* (bacterial), *↑ Ampelomyces* sp. IRAN_1 (fungal) White-thick (W-thick): *↑ Megasphaera micronuciformis, Selenomonas sputigena* ATCC 35185, *Acinetobacter ursingii, Prevotella maculosa* Yellow-thick (Y-thick): *↑ Blastobotrys adeninivorans* (fungal) Yellow-thin (Y-thin): no significantly increased taxa with AUC >0.6
Wu et al.^27^ China	Gastric Adenocarcinoma Case-control; N = 57/80 (GC /healthy controls)	Sterile swab (midline of tongue dorsum, 3 times) 16S rRNA (V4 region) Roche Genome Sequencer FLX+ (pyrosequencing)	Age, gender, smoking, drinking Oral health status, HP infection status, antibiotics/PPI use (excl. 2 weeks only), diet, BMI, tea consumption	Enriched in GC: Firmicutes (phylum), *Streptococcus, Abiotrophia* Depleted in GC: Bacteroidetes (phylum), *Neisseria, Prevotella, Prevotella7, Porphyromonas* Cardia-specific enriched: *Abiotrophia, Alloprevotella, Veillonella* Non-cardia enriched: *Streptococcus, Ochrobactrum, Achromobacter*
Lu et al.^28^ China	Liver Carcinoma (Hepatocellular carcinoma) with cirrhosis Case-control; N = 35/25 (LC with cirrhosis/healthy controls)	Tongue scraper (posterior to anterior middle area) 16S rRNA (V3-V4) Illumina MiSeq 2000	Age, gender, BMI, no periodontitis/gingivitis, antibiotics/probiotics (excl. 8 weeks) Smoking, drinking, diet, PPI use, tea consumption, oral hygiene habits (brushing frequency)	Enriched in LC: *Fusobacteria, Actinobacteria, SR1; Fusobacterium, Leptotrichia, Actinomyces, Campylobacter, Oribacterium* Depleted in LC: Bacteroidetes (phylum); *Haemophilus, Streptococcus, Pseudomonas*
Hu et al.^29^ China	Gastric Cancer Case-control, N = 74/72; subset sequenced: 34 GC /17 HC	Sterile swab (tongue surface, 3 swabs × 3 times) 16S rRNA (V2-V4) Illumina MiSeq	Age, gender, BMI, smoking, drinking, diabetes, hypertension Antibiotics (excl. 2 months only), PPI use, oral hygiene status, diet, tea consumption, *HP* infection status, tumour stage/site	Enriched in GC: Actinobacteria (phylum), *Actinomyces, Streptococcus* Depleted in GC: Proteobacteria (phylum), *Fusobacterium, Neisseria, Haemophilus, Porphyromonas* Thick coating vs. thin coating: Lower microbial diversity, higher *Actinomyces* and *Streptococcus*
Han et al.^30^ China	Colorectal Cancer Case-control, N = 45/47; subset sequenced: 14 CRC /7 HC	Tongue surface (unspecified exact location, likely entire dorsal surface) 16S rRNA (V2-V4) Illumina MiSeq	Age, gender, BMI, smoking, diabetes, hypertension Antibiotic use, PPI use, oral hygiene status, diet, tea consumption, *HP* infection status, tumour stage/site, chemotherapy/surgery	Thick coating vs. control: Higher *Prevotella, Leptotrichia, Actinomyces;* Lower *Gemella, Acinetobacter, Brochothrix, Chryseobacterium, Flavobacterium, Myroides, Parvimonas, Yersinia* Thin coating vs. control: Higher *Enhydrobacter, Janthinobacterium, Rahnella;* Lower *Veillonella* CRC group overall: Higher *Streptococcus;* Lower *Haemophilus*

HC, Healthy controls; CRC, Colorectal cancer; CRA, Colorectal adenoma; GC, Gastric cancer; EAC, Oesophageal adenocarcinoma; PLC, Primary liver cancer; PHC, Pancreatic head carcinoma; DST, Digestive system tumours; OSCC, Oral squamous cell carcinoma; PSM, Propensity score matching; PPI, Proton pump inhibitor; BMI, Body mass index; ASV, Amplicon sequence variant; AUC, Area under the curve.

This table summarises studies evaluating tongue-derived microbiome profiles in gastrointestinal and related malignancies, including colorectal, gastric, oesophageal, pancreatic and liver cancers. Data include study design, population characteristics, tongue sampling methodology, sequencing platforms, confounders addressed and direction of microbial changes relative to controls or disease subgroups.

Key Pattern Identified: Across gastrointestinal malignancies, tongue-derived microbial profiles consistently demonstrate enrichment of anaerobic and pro-inflammatory taxa alongside depletion of health-associated commensals. These changes are accompanied by increased diversity and disease-specific microbial restructuring, although variability across cancer types and lack of confounder adjustment limit consistency of directionality for individual taxa.

**Table 3:** Tongue-derived multi-omics signatures in gastrointestinal disease: metabolomic, lipidomic, transcriptomic and proteomic findings.

Metabolomics/Lipidomics							
Study (Year)Country	Cancer type designN=(case/control)	Histology confirmedTongue sampling	Omics platformMetabolite database	# MetabolitesMajor classes	Effect direction	Diagnostic model	Pathological correlations
You et al.^31^ China	Gastric precancerous lesions (chronic gastritis, atrophy, intestinal metaplasia) Cross-sectional (case-control) N = 350 CG/50 HC	Gastroscopy + gastric mucosal pathology Tongue coating scraping; GC-TOF-MS + UHPLC-QE-MS	Metabolomics GC-TOF-MS + UHPLC-QE-MS (positive & negative) LECO-Fiehn Rtx5 (GC); HMDB, KEGG, PubChem (LC)	101 total (54↑, 47↓) Lipids/lipid-like (47), organic acids^13^	↑: 4-Ipomeanol, Nervonic acid, Sphinganine 1-P; ↓: 1-Methyladenosine, 3-Hydroxycapric acid	5 metabolites: accuracy 95.4%, specificity 87.4%, sensitivity 88.0%	Atrophy,^10^ IM,^10^ Hp infection^3^
Chen et al.^34^ China	Colorectal cancer + adenoma (CRA, precancer) Cross-sectional (case-control) N = 92 (32 CRC/30 CRA/30 HC)	Colonoscopy + postoperative pathology (CRA/CRC); colonoscopy (HC) Tongue coating scraping (microfibre brush, 20 rotations); UHPLC-Q Exactive Plus MS lipidomics	Lipidomics UHPLC-Q Exactive Plus MS (positive & negative) MS-DIAL v5.1 + MassBank	21-lipid panel (CRC vs HC); 9-lipid panel (CRC vs CRA) Long-chain unsaturated TGs, NAE, LPC, LPE, CAR (↑); PE-P, MUFA-PE, PG, LNAPE, PE-Cer, SM, NAOrn, 12-HETE (↓)	↑ CRC: long-chain unsaturated TGs, NAE, LPC, LPE, CAR, FA(18:2), FA(18:3); ↓ CRC: PE-P, MUFA-PE, PG, LNAPE, PE-Cer, SM, NAOrn, oxidised FA(18:1;O2), 12-HETE	21 lipids: AUC = 0.89 (CRC vs HC); 9 lipids: AUC = 0.90 (CRC vs CRA)	TG(52:2) positively, PE(P-29:1) negatively correlated with adenoma-carcinoma progression; NAOrn, PE-Cer, SM decreased with progression
Hao et al.^14^ China	Gastric precancerous lesions (GPL) – intestinal metaplasia/dysplasia Cross-sectional (case-control) N = 75 (60 GPL/15 HC)	Gastroscopy + gastric mucosal pathology Tongue coating scraping (sterile swab, 5 strokes); GC-TOF-MS + UHPLC-QE-MS	Metabolomics GC-TOF-MS + UHPLC-QE-MS (positive & negative) GC: LECO-Fiehn Rtx5; LC: in-house MS2 (BiotreeDB V2.1)	134 total (133↑, 1↓) Lipids/lipid-like (71, 53.38%); organoheterocyclic^12^; organic acids^11^	↑ in GPL: sphinganine 1-phosphate, leukotriene D4, prostaglandin D2, sarcosine, most lipids; ↓: lactosylceramide (d18:1/26:0)	Pathways: sphingolipid metabolism, arachidonic acid metabolism, glycine/serine/threonine metabolism, valine/leucine/isoleucine degradation (no ROC model)	Not explicitly reported
Transcriptomics/Metatranscriptomics							
Study (Year) Country	Cancer Type Design N=(case/control)	Histology Confirmed	Tongue Sampling	Transcriptomics Platform	Host vs. microbial transcripts	Number of differentially expressed genes/transcripts	Pathway enrichment

Jain et al.^33^ USA	Oral Squamous Cell Carcinoma Cross-sectional (case-control) N = 60 (20 OSCC tumour/20 adjacent normal/20 HC)	histopathologic evaluation of frozen sections; HPV16/18-negative by PCR)	Tissue: Laser microdissection (LMD) of frozen OSCC tumour & adjacent normal epithelium (mobile tongue). Healthy: Deep epithelial tongue scrapings (dermatologic curette)	Ultra-deep metatranscriptome sequencing (RNA-seq) DNBSEQ-T7 platform, 2 × 100 bp CoolMPS chemistry ∼400 million paired reads/sample	Host: mapped to GRCh38; Microbial: unmapped reads processed with HUMAnN 3.0	Host DEGs (FDR ≤0.05, fold change ≥2): • TT vs. ANT: 8640 (5186↑, 3454↓) • TT vs. HC: 17,991 (10,400↑, 7591↓) • ANT vs. HC: 15,375 (8436↑, 6939↓) Microbial differentially abundant taxa: • 36 bacterial genera enriched in HC (e.g., *Gemella, Prevotella, Fusobacterium*) • OSCC-enriched: *Cutibacterium acnes, Malassezia restricta, Human Herpes Virus 6B, Yuavirus*, etc.	Host (GSEA, Hallmark gene sets): • TT vs. ANT: ↑ EMT, inflammatory response, KRAS, IFN responses; ↓ oxidative phosphorylation, fatty acid metabolism, P53 • TT/ANT vs. HC: ↑ E2F targets, MYC targets, G2-M checkpoint, EMT; ↓ heme metabolism, P53, apoptosis Microbiome-host integration: OSCC-associated taxa (*Cutibacterium, Roseolovirus, Yuavirus*) correlated with upregulation of proliferation pathways (E2F targets, MYC targets, G2-M checkpoint)

Proteomics							
Study (Year)Country	Cancer typeDesignN=(case/control)	Histology ConfirmedTongue sample	Proteomics platform	Protein database	Number of proteins identified	Proteins of interest	Functional pathway mapping
Chen et al.^34^ China	Gastric adenocarcinoma (stomach or gastro-oesophageal junction) Cross-sectional multicentre diagnostic study, N = 365 (180 GC /185 non-GC) multi-centre (5 centres)	histologically confirmed adenocarcinoma of the stomach or gastro-oesophageal junction Tongue coating swab (root to tip × 15 with rolling motion, 3 swabs total)	PCT-DIA (Pressure Cycling Technology – Data Independent Acquisition) mass spectrometry	Custom-built: metagenomic sequencing of 20 samples (healthy, gastritis, stage I-II, stage III-IV) + UniProt human database (UP000005640)	Total: 15,212 (Human: 1432; Microbial: 13,780)	Downregulated in GC (both cohorts): KRT2, KRT9, DCD, EWSR1, CACNA1G, COG1136 (ABC transporter). GC risk-associated bacteria: *Schaalia odontolytica* (ZJC), *Neisseria brasiliensis* (multi-centre)	Human downregulated: Growth/differentiation of tongue epithelium (weakened physical barrier). Microbial downregulated: Biosynthesis of cofactors, oxidative phosphorylation and Amino acid biosynthesis. Microbial upregulated: Two-component system, carbon metabolism, butanoate metabolism. Top 50 microbial protein model: Ribosome pathway (AUC = 0.91 independent validation)

HC, healthy controls; CG, chronic gastritis; GPL, gastric precancerous lesions; CRC, colorectal cancer; CRA, colorectal adenoma; OSCC, oral squamous cell carcinoma; TG, triglyceride; LPC, lysophosphatidylcholine; LPE, lysophosphatidylethanolamine; NAE, N-acylethanolamine; CAR, carnitine; PE, phosphatidylethanolamine; PE-P, plasmalogen phosphatidylethanolamine; MUFA-PE, monounsaturated fatty acid phosphatidylethanolamine; PG, phosphatidylglycerol; LNAPE, N-linoleoyl phosphatidylethanolamine; PE-Cer, phosphoethanolamine ceramide; SM, sphingomyelin; NAOrn, N-acetylornithine; HETE, hydroxyeicosatetraenoic acid; FA, fatty acid; IM, intestinal metaplasia; Hp, Helicobacter pylori; GC-TOF-MS, gas chromatography–time-of-flight mass spectrometry; UHPLC-QE-MS, ultra-high-performance liquid chromatography–Q Exactive mass spectrometry; HMDB, Human Metabolome Database; KEGG, Kyoto Encyclopedia of Genes and Genomes; MS-DIAL, Mass Spectrometry–Data Independent AnaLysis; DEG, differentially expressed gene; GSEA, Gene Set Enrichment Analysis; EMT, epithelial–mesenchymal transition; FDR, false discovery rate; KRAS, Kirsten rat sarcoma viral oncogene homolog; IFN, interferon; MYC, MYC proto-oncogene; PCT-DIA, pressure cycling technology–data independent acquisition; ROC, receiver operating characteristic; AUC, area under the curve; TBA, total bile acids.

This table summarises studies investigating functional and multi-omics alterations in tongue-derived samples across gastrointestinal disease states. Included modalities encompass metabolomics, lipidomics, transcriptomics and proteomics, with emphasis on major molecular classes, direction of change, diagnostic model performance and pathological correlations.

Key Pattern Identified: Across metabolomic, lipidomic, proteomic and transcriptomic studies, tongue-derived multi-omics signatures consistently highlight lipid metabolism as a dominant altered pathway, alongside changes in inflammatory signalling and epithelial barrier function. These functional alterations parallel microbial restructuring and suggest convergence of biological pathways across gastrointestinal disease stages.

**Table 4:** Interpretive synthesis and evidence confidence framework for tongue-derived microbial and multi-omics signatures across gastrointestinal disease stages.

Study (Year)Country	GI disease	Interpretive takeaway	Evidence confidence
GI Inflammatory + Precancer			
Yanan et al.^10^ China	Chronic gastritis	Mild, non-specific dysbiosis with increased diversity; strong phenotype dependence but poor confounder control limits disease-specific interpretation.	Low
Xiao et al.^12^ China	Gastric precancer	Early anaerobe-associated restructuring with phenotype-linked variation; findings suggest emerging dysbiosis but lack robustness due to incomplete confounder adjustment.	Moderate
Xiao et al.^12^ China	Oesophageal precancer	Network disruption with preserved diversity indicates ecological instability rather than compositional collapse; signal likely influenced by unadjusted lifestyle factors.	Moderate
Kang et al.^13^ China	Precancerous lesions of the upper gastrointestinal tract	Host–microbial interaction signals (IL-17A correlations) suggest potential immune-microbiome coupling, but causal direction remains unclear due to cross-sectional design.	Moderate
Hao et al.^14^ China	Gastric precancerous lesion (multi-omics)	Strong convergence towards lipid metabolic dysregulation supports functional microbiome–host coupling in precancerous states.	Moderate
Cui et al.^15^ China	Gastritis cascade	Gradient-like microbial transitions across disease stages suggest progressive ecological restructuring, but findings remain population-specific.	Moderate
		Inflammatory GI disease (overall): Mild, non-specific dysbiosis; insufficient evidence for disease specificity Precancer (overall): Emerging anaerobe-enriched restructuring with phenotype-dependent modulation	Low Moderate
GI Malignancy			
Liu et al.^16^ China	CRC	Progressive ecological shift across adenoma–carcinoma sequence supports disease-stage association but remains confounded by unmeasured oral and medication factors.	Moderate
Ma et al.^17^ China	Digestive System Tumour cohort	A large cohort supports reproducible anaerobic enrichment and commensal depletion across GI cancers, strengthening the consistency of the malignant signature.	Higher
Wang et al.^18^ China	Oesophageal adenocarcinoma	Distinct dysbiosis with increased diversity suggests tumour-associated ecological restructuring rather than simple loss of taxa.	Moderate
Zheng et al.^19^ China	Colorectal Cancer	Strong phenotype–microbiome linkage highlights coating classification as a key modifier, but the absence of controls limits interpretability.	Low
Chen et al.^34^ China	Colorectal Cancer	Consistent depletion of commensals and enrichment of opportunistic taxa reinforces the malignant dysbiosis pattern across sequencing platforms.	Moderate
Nie et al.^21^ China	*OSCC (multi-niche comparison)	Multi-site comparison demonstrates strong enrichment of anaerobic and pathogenic taxa in tumour-associated niches, supporting localised tumour–microbiome interactions, but absence of healthy controls and lack of confounder adjustment severely limit interpretability.	Low
Zhang et al.^22^ China	Liver cancer	Distinct microbial signatures with phenotype stratification suggest potential discriminatory models, though confounding limits biomarker claims.	Moderate
Chung et al.^23^ USA	Pancreatic	Shared oral–gut taxa support the biological plausibility of the oral–gut axis but do not establish directionality or causality.	Moderate
Xu et al.^24^ China	Gastric cancer	Integrated microbial–immune–metabolic alterations suggest microbial–immune interaction, but the absence of validation limits translational value.	Moderate
Lu et al.^25^ China	Pancreatic head carcinoma	Marked enrichment of anaerobic and opportunistic taxa with depletion of commensals suggests tumour-associated ecological restructuring, but small sample size and incomplete confounder adjustment limit robustness.	Moderate–Low
Xu et al.^26^ China	Gastric cancer	Strong phenotype-associated microbial stratification highlights tongue coating classification as a key modifier of microbial (including fungal) signals, but heterogeneity and lack of standardised phenotype taxonomy limit comparability across studies.	Moderate
Wu et al.^27^ China	Gastric adenocarcinoma	Consistent phylum-level shifts with commensal depletion and opportunistic enrichment support malignant dysbiosis, but limited sequencing depth and incomplete confounder control reduce interpretive strength.	Moderate–Low
Lu et al.^25^ China	Liver carcinoma	Clear enrichment of anaerobic and inflammation-associated taxa supports malignant ecological restructuring, but early-generation sequencing and limited confounder control restrict generalisability.	Moderate–Low
Hu et al.^29^ China	Gastric cancer	Early evidence of commensal depletion and Actinomyces/Streptococcus enrichment suggests tumour-associated dysbiosis, but subset sequencing and incomplete confounder adjustment weaken conclusions.	Moderate–Low
Han et al.^30^ China	Colorectal cancer	Preliminary evidence of coating-dependent microbial shifts suggests phenotype–microbiome interaction, but very small sample size and lack of confounder adjustment severely limit reliability.	Low
		Malignancy (overall): More consistent ecological restructuring with commensal depletion and anaerobe enrichment; the strongest signal, but still confounded	Moderate–Higher
Multi-omics			
You et al.^31^ China	Gastric precancerous	High diagnostic performance driven by lipid metabolites suggests a strong functional signal, but a lack of validation limits clinical applicability.	Moderate
Chen et al.^20^ China	CRC + colorectal adenoma	Reproducible lipid panel distinguishes CRC from precancer and controls, supporting metabolomic progression along adenoma–carcinoma axis.	Moderate–Higher
Hao et al.^14^ China	Gastric precancer	Dominance of lipid pathways reinforces metabolic convergence across studies, indicating functional dysbiosis rather than taxonomic specificity.	Moderate
Jain et al.^33^ USA	*OSCC	Strong host–microbial transcriptomic coupling highlights mechanistic links to oncogenic pathways, but tissue vs. coating heterogeneity complicates interpretation.	Moderate
Chen et al.^20^ China	Gastric adenocarcinoma	Proteomic alterations support functional host–microbial interaction and pathway-level dysregulation, reinforcing multi-omics convergence, but limited external validation constrains translational interpretation.	Moderate
		Multi-omics (overall): Lipid metabolism, inflammatory signalling and epithelial barrier pathways show consistent functional convergence across disease states	Moderate

CAG, chronic atrophic gastritis; CG, chronic gastritis; CRC, colorectal cancer; CRA, colorectal adenoma; DST, digestive system tumours; EAC, oesophageal adenocarcinoma; GPL, gastric precancerous lesions; GI, gastrointestinal; IM, intestinal metaplasia; OSCC, oral squamous cell carcinoma; PHC, pancreatic head carcinoma; PLC, primary liver cancer; PLUGT, precancerous lesions of the upper gastrointestinal tract; PPI, proton pump inhibitor.

Studies are grouped into three categories: inflammatory/precancerous gastrointestinal conditions, gastrointestinal malignancies and multi-omics investigations. An overall synthesis is provided for each category to highlight cross-study consistency and key biological patterns. *Studies involving oral squamous cell carcinoma (OSCC) are included for mechanistic comparison and are not classified as gastrointestinal disease.

*Low:* Cross-sectional studies with small sample sizes and limited confounder control; *Moderate:* Multiple studies showing consistent findings but lacking external validation; *Higher:* Reproducible findings across larger cohorts with partial confounder adjustment.

Interpretations should be considered within the context of predominantly cross-sectional study designs, substantial heterogeneity in methodology, and frequent lack of adjustment for key confounders, including proton pump inhibitor use, diet, oral hygiene and medication exposure. Accordingly, findings represent associative biological signals rather than validated diagnostic biomarkers.

## Results

Database searching identified 742 records, with 6 additional records identified manually. Following the removal of 221 duplicates, 527 records underwent title and abstract screening, resulting in the exclusion of 468 records. Fifty-nine full-text articles were assessed for eligibility, of which 34 were excluded with reasons (no tongue sampling [n = 14]; saliva/plaque only [n = 8]; animal studies [n = 6]; review/commentary [n = 3]; no molecular analysis [n = 3]). Ultimately, 25 studies^10^, ^11^, ^12^, ^13^, ^14^, ^15^, ^16^, ^17^, ^18^, ^19^, ^20^, ^21^, ^22^, ^23^, ^24^, ^25^, ^26^, ^27^, ^28^, ^29^, ^30^, ^31^, ^32^, ^33^, ^34^ met the inclusion criteria and were included in the final scoping synthesis. Twenty-three studies evaluated gastrointestinal conditions, while 2 additional studies on OSCC were retained as oral mechanistic comparators. A defining characteristic of the evidence base is its geographic concentration: 23/25 studies (92%) were conducted in East Asian populations (predominantly China), with only two studies from the USA.^23^^,^^33^ All included studies employed cross-sectional designs, either case-control or cross-sectional comparative. No longitudinal studies tracking intra-individual tongue microbiome dynamics over time were identified, and none validated their findings in an independent external cohort.

Three consistent patterns emerged across studies: (1) stage-associated microbial restructuring, (2) convergence of multi-omics signals, with a particular emphasis on lipid metabolism and (3) phenotype-associated modification of microbial and functional profiles.

Across all studies, more than 4500 participants were included, with individual sample sizes ranging from 14 to 1199. Because several studies originated from overlapping research groups and institutions; participant counts may not represent unique individuals. Therefore, the cumulative sample size (>4500 participants) should be interpreted as an approximate upper-bound estimate. Larger studies primarily examined cancer cohorts, whereas smaller studies more frequently investigated precancer populations.

Tongue-derived samples were primarily collected from the dorsal tongue surface or tongue coating using swabs, scrapers, or standardised toothbrushes. Sampling was typically performed under fasting or morning conditions prior to oral hygiene. Study characteristics, including design, population, analytical platform and addressed confounders, are summarised in Table 1, Table 2, Table 3.

### Tongue signals in inflammatory GI disease

The available evidence linking the inflammatory GI disease spectrum and tongue signals remains limited, as only one primary microbiome study^10^ and one metabolomics investigation have been conducted.^31^ In cases of chronic gastritis, tongue coating dysbiosis was marked by an increased presence of *Fusobacterium* and altered levels of *Neisseria* and *Prevotella*, along with higher alpha diversity compared to healthy controls. Microbial profiles derived from tongue samples showed minimal differentiation by inflammation severity.

Complementary metabolomic analysis identified 101 differential metabolites, with more than 50% classified as lipid and lipid-like molecules,^31^ suggesting early functional changes at this stage. Inflammatory gastrointestinal disease was associated with mild, non-specific alterations in tongue-derived microbial and metabolic profiles (Table 1).

### Tongue-derived dysbiosis in GI precancer

Six studies assessed tongue-derived microbial signatures in precancerous gastrointestinal conditions, specifically gastric intestinal metaplasia, dysplasia and oesophageal precancer (Table 1).

#### Upper gastrointestinal and oesophageal precancer

Studies of upper gastrointestinal precancer consistently reported compositional shifts.^10^^,^^12^^,^^13^ These shifts included enrichment of anaerobic taxa such as *Parvimonas, Peptostreptococcus* and *Capnocytophaga,* with preservation of alpha diversity, and changes in microbial network structure.

Host-microbial associations were also reported, including correlations between *Parvimonas* and inflammatory markers such as IL-17A.^13^

#### Gastric precancer

Investigations of gastric-precancerous lesions demonstrated enrichment of anaerobic and inflammation-associated taxa, such as *Solobacterium, Rothia, Alloprevotella* and *Oribacterium.*^11^^,^^14^^,^^15^

Multi-omics analyses indicated that lipid-related metabolites comprised over 50% of differential metabolites, with frequent involvement of lipid-associated pathways.^14^

#### Precancer as a progressive dysbiosis gradient

A single-cohort study^15^ demonstrated a progressive microbial gradient across the gastritis-to-precancer cascade, with directional changes in taxa abundance observed across disease stages.

#### Directional inconsistencies in precancer studies

Across these studies, enrichment of anaerobic taxa and preservation of alpha diversity were common findings. However, the directionality of specific taxa varied across studies, likely due to differences in disease stage, anatomical site, tongue phenotype classification and sequencing platforms.

### Phenotype-associated stratification of tongue signals

Multiple studies have demonstrated that tongue coating phenotype, including colour, thickness and classification, influences microbial composition and functional outputs across various disease states.

In upper gastrointestinal precancer, yellow tongue coatings were associated with increased microbial diversity and enrichment of taxa such as *Leptotrichia, Campylobacter* and *Parvimonas,* whereas white coatings exhibited distinct compositional profiles.^13^ Thick coatings were linked to altered microbial abundance compared to thin coatings.

In gastric cancer cohorts, distinct microbial patterns were identified according to tongue coating phenotype, with differential bacterial and fungal enrichment observed between white-thick and yellow-thick coatings.^26^

Additional studies have reported variation in microbial composition, diversity and host-microbial correlations across tongue coating phenotype categories, including associations with metabolic and lifestyle factors such as bile acid profiles.^12^^,^^22^

However, tongue phenotype classification systems were not standardised across studies (e.g., white/yellow, thin/thick, greasy/peeling), which limited direct comparisons between cohorts. Most phenotype classifications relied on visual inspection rather than quantitative measurement, such as colorimetry or coating thickness quantification, thereby introducing observer variability.

Overall, differences in microbial composition and functional profiles associated with tongue coating phenotype were consistently observed across various disease categories.

### Tongue-derived signals in gastrointestinal malignancy

Fifteen studies have evaluated changes in the tongue-derived microbiome associated with gastrointestinal malignancies (Table 2). In gastric, colorectal, oesophageal, pancreatic and liver cancers, tongue coating biofilms consistently exhibited ecological restructuring. Frequently reported patterns included depletion of commensal taxa such as *Neisseria, Haemophilus* and *Rothia*, alongside enrichment of anaerobic and opportunistic organisms including *Prevotella, Fusobacterium, Actinomyces* and *Leptotrichia*.

Compared to inflammatory and precancerous conditions, malignant cohorts demonstrated greater consistency in microbiome alterations across studies and showed clear differentiation from healthy controls.

These findings suggest that malignancy is associated with reproducible alterations in microbial composition in malignancy, specifically characterised by depletion of commensal taxa and enrichment of anaerobic organisms.

### Multi-omics signatures in gastrointestinal disease

Across metabolomic, lipidomic, proteomic and transcriptomic studies, consistent functional alterations were identified.

Lipid metabolism emerged as the primarily affected pathway, with diverse lipid-related metabolites and analogous molecules identified across several studies. Common perturbed pathways included sphingolipid metabolism, arachidonic acid metabolism and lipid signalling (Table 3).

Proteomic analyses^34^ revealed changes in proteins associated with the epithelial barrier and microbial functional pathways. Transcriptomic studies identified alterations in host gene inflammation, cellular proliferation and metabolic signalling.

The number of multi-omics studies was limited (n= 5), and independent replication was not reported. Nevertheless, multi-omics findings consistently demonstrated functional alterations across disease stages, particularly relation to lipid metabolism and host-microbiome interactions.

## Discussion

This scoping review offers an integrative synthesis of tongue coating microbiome and multi-omics associations within an oral-systemic framework, with particular emphasis on inflammatory, precancerous, and malignant gastrointestinal conditions. In addressing its objectives, the review identifies: (1) stage-associated microbial variations across diseases; (2) convergence of multi-omics findings on lipid metabolism across cohorts; and (3) microbial and functional shifts associated with tongue coating phenotypes.

This review advances the field by integrating microbial, functional and phenotype-dependent findings into a unified interpretive framework, distinguishing reproducible biological patterns from limitations imposed by study design and confounding factors. In contrast to individual primary studies that report isolated taxonomic associations, this review contextualises tongue-derived signals within ecological and methodological constraints. It is clearly indicated that the observed profiles present associations rather than causative relationship.

A recent review by Yamauchi et al.^35^ summarised tongue microbiome associations with gastrointestinal disease. The present review extends these works by integrating microbial, metabolomic, lipidomic, transcriptomic and proteomic evidence within a unified phenotype-microbiome-metabolome framework, while explicitly evaluating confounding, biomarker readiness, and methodological limitations.

### Microbial patterns across disease stages

Across the included studies, variations in microbial composition were observed among inflammatory, precancerous and malignant conditions. In inflammatory states, alterations were generally mild and non-specific, characterised by modest compositional shifts and increased diversity.^10^^,^^36^ These changes did not demonstrate clearly discriminatory capacity between diseased and healthy states, indicating limited specificity at this stage.

In precancerous lesions, microbial profiles were characterised by enrichment of anaerobic and inflammation-associated taxa.^11^, ^12^, ^13^^,^^15^ Alpha diversity was frequently preserved, with concurrent changes in microbial network structure reported across studies.

In malignant conditions, more consistent compositional patterns were observed across studies, including depletion of commensal taxa and enrichment of anaerobic and opportunistic organisms.^17^^,^^20^^,^^24^ These findings suggest greater reproducibility of microbial alterations in advanced disease compared with earlier stages.

**Fig. 1:**
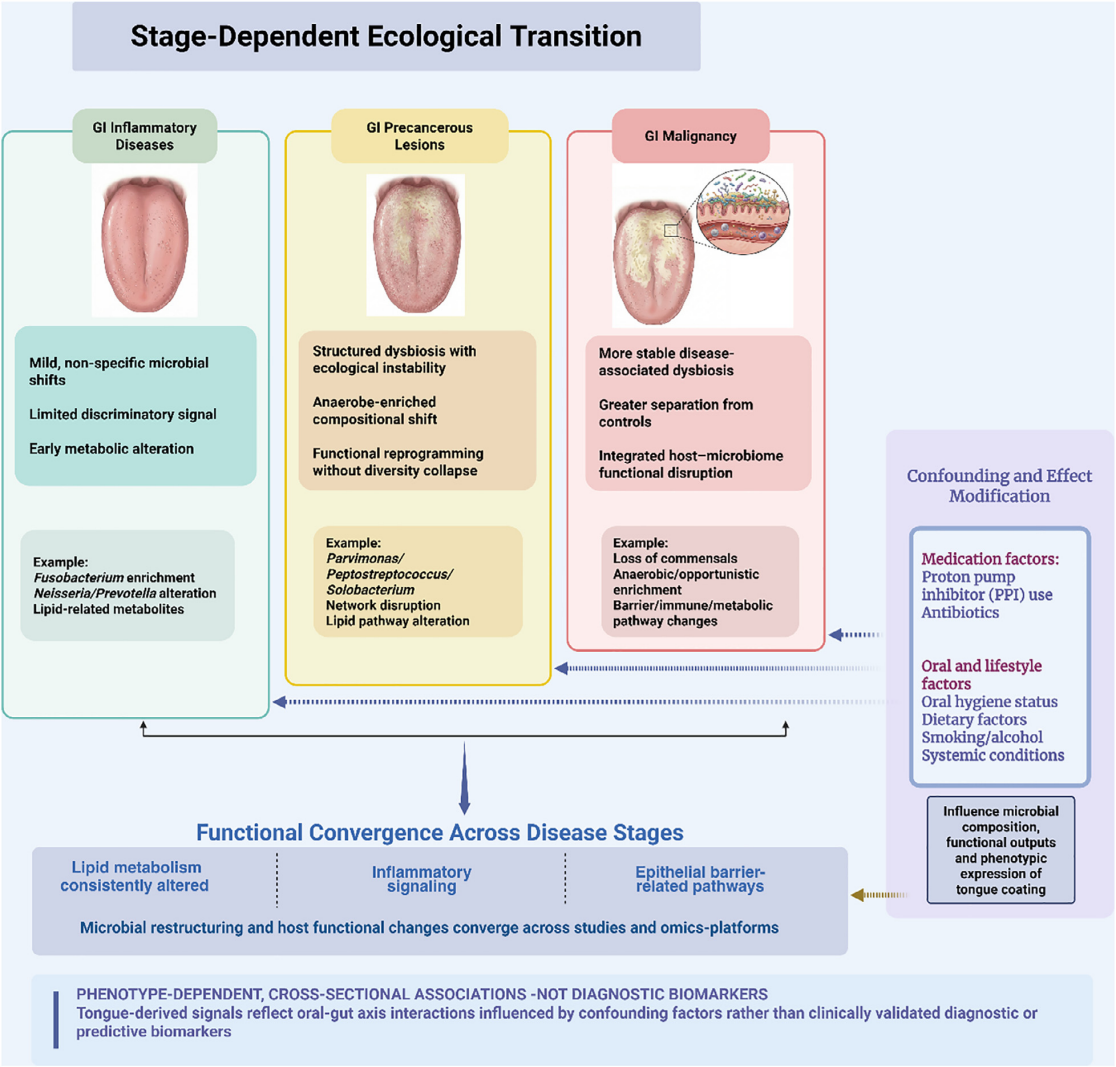
Conceptual model of tongue-derived microbial and multi-omics signals across gastrointestinal disease stages. This schematic integrates cross-sectional evidence demonstrating stage-associated differences in tongue-derived biological signals across inflammatory, precancerous and malignant gastrointestinal conditions. Microbial profiles transition from mild, non-specific alterations in inflammatory states to structured, anaerobe-enriched dysbiosis in precancer, and to more stable ecological restructuring in malignancy. Across all stages, multi-omics analyses consistently converge on lipid metabolism, inflammatory signalling and epithelial barrier-related pathways, suggesting functional coupling between microbial and host processes. Tongue coating phenotype (colour, thickness and classification) acts as a key effect modifier influencing both microbial composition and functional outputs. All observations derive from cross-sectional comparisons and therefore represent associations rather than temporal progression or causality. Accordingly, tongue-derived signals should be interpreted as exploratory, hypothesis-generating indicators of oral–gut axis interactions rather than clinically applicable diagnostic biomarkers. All graphical elements, including the tongue illustrations, were created with BioRender.com.

Taken together, these observations indicate that tongue-derived microbial profiles vary across disease categories, with increasing consistency of compositional changes observed in malignancy. These stage-associated patterns can be conceptualised as progressive ecological restructuring from inflammatory to malignant states rather than as evidence of confirmed temporal progression (Figure 1).

### Functional convergence of multi-omics signals

In addition to compositional changes, multi-omics analyses offer provide functional context that corroborates microbial findings. Metabolomic and lipidomic studies consistently report that lipid and lipid-like molecules constitute a significant proportion of differential metabolites across independent cohorts.^14^^,^^20^^,^^31^

Frequently identified pathways included sphingolipid metabolism, arachidonic acid metabolism and lipid signalling. Proteomic and transcriptomic studies also reveal changes in epithelial barrier-associated processes and inflammatory signalling pathways.^14^^,^^31^

The consistent identification of these functional pathways across independent cohorts indicates convergence at the pathway level rather than at the level of individual taxa. These findings suggest that tongue-derived signals represent coordinated host-microbiome functional alterations in gastrointestinal disease states.

### Phenotype as a biological effect modifier

A consistent finding across studies is the influence of tongue coating phenotype on microbial composition and functional outputs. Variations in coating colour, thickness and classification systems were associated with differences in microbial diversity and taxonomic composition across both precancerous and malignant conditions.^13^^,^^22^^,^^29^

Phenotype-associated differences were reported in multiple studies, regardless of disease category, with distinct microbial patterns linked to specific coating characteristics. However, phenotype classification systems varied considerably across studies, such as white versus yellow, thin versus thick and greasy versus peeling, which limits comparability between cohorts. These observations point to the fact that tongue phenotype is a significant modifier of observed microbial and functional signals and should be incorporated into study design and analysis.

### Methodological limitations and confounding

Interpretation of these findings is constrained by several methodological constraints (Table 4). Notably, all included studies utilised cross-sectional designs, which capture only single time points and preclude conclusions regarding temporal progression or causality. Consequently, observed differences between disease stages reflect comparisons across independent cohorts rather than longitudinal changes within individuals.

#### Confounding as a central interpretive constraint

Across included studies, the most frequent unaddressed or partially addressed confounders were proton pump inhibitor use, oral hygiene practices, dietary patterns, antibiotic exposure windows, periodontal status, smoking, alcohol use and cancer treatment history. These variables are biologically relevant because each can independently alter tongue biofilm composition, oral microbial load, salivary ecology, gastric acidity and gastrointestinal microbial exposure.

For instance, proton pump inhibitors exert broad, system-level effects on the gastrointestinal tract by chronically suppressing gastric acid secretion. This loss of the gastric acid barrier permits survival and distal translocation of ingested and oral microbes, promoting ectopic colonisation by oral commensals within the intestinal microbiome, reduced microbial diversity and dysbiosis characterised by expansion of opportunistic organisms.^37^^,^^38^ Functionally, these shifts are accompanied by altered bile acid metabolism, reduced short-chain fatty acid production and impaired colonisation resistance, collectively increasing susceptibility to enteric infections and small intestinal bacterial overgrowth.^39^ At the mucosal level, PPIs may contribute to epithelial barrier perturbation and immune modulation, with downstream effects on inflammation and nutrient absorption.^40^^,^^41^ Importantly, these changes reflect a phenotype-microbiome-metabolome shift within the gastrointestinal ecosystem.

In the absence of stratification or multivariable adjustment for PPI use, diet, oral hygiene, periodontal status and recent antibiotic exposure, disease-specific interpretation of tongue-derived signals remains premature.

#### Geographic concentration and external validity

The geographic concentration of the evidence represents another significant limitation for translational interpretation. Most included studies were conducted in East Asian populations, particularly in China. While this distribution is understandable due to the historical and clinical relevance of tongue diagnosis in traditional Chinese medicine, it restricts generalisability.

Tongue microbiome composition is shaped by numerous context-specific factors, including diet, ethnicity, geography, oral hygiene practices, fermented food intake, tea consumption, medication patterns and healthcare-seeking behaviour. As a result, findings derived primarily from East Asian cohorts cannot be directly extrapolated to Western, Middle Eastern, African, or multi-ethnic populations. Future validation studies should incorporate geographically diverse cohorts and assess whether reported tongue-derived signatures remain robust after adjustment for diet, ethnicity, medication use, oral hygiene and socioeconomic variables.

### Interpretive framework: competing explanatory models

Within these methodological constraints, several explanatory models warrant consideration.

First, a *causal model* posits that tongue-derived microbial communities contribute directly to gastrointestinal disease, possibly through mechanisms such as microbial translocation or systemic inflammatory signalling. While biologically plausible, this hypothesis remains unsubstantiated in the absence of longitudinal or mechanistic evidence.

Second, a *reactive model* proposes that gastrointestinal disease and its associated treatments influence tongue microbiota as a downstream effect. This interpretation is consistent with the limited adjustment for treatment exposure and other clinical variables across the included studies.

Finally, a *confounded model* suggests that shared external factors independently influence both tongue microbiota and disease risk. Given the frequent lack of rigorous control for confounders, this explanation cannot be excluded and is likely to contribute to at least part of the observed associations.

Taken together, these models highlight the interpretive limitations inherent in cross-sectional data and emphasise the necessity for caution, non-causal framing of current findings.

### Translational positioning: what can and cannot be inferred clinically

Tongue examination remains an established component of dental and oral medicine practice for the assessment of local mucosal pathology, coating burden, candidal overgrowth, halitosis-related biofilm accumulation and oral hygiene status.

From a systemic diagnostic perspective, current evidence does not support the use of tongue-derived microbial or multi-omics signatures as clinical biomarkers for gastrointestinal disease. At present, these signals should be regarded as research tools with potential for hypothesis generation, exploratory disease stratification and investigation of oral-gut interactions.

Translation into clinical applications will require longitudinal cohort studies with intra-individual follow-up, standardised tongue sampling protocols, reproducible phenotype classification systems and rigorous adjustment for oral and systemic confounders. Integration of multi-omics data with clinical behavioural and lifestyle variables will be essential to determine whether tongue-derived signatures offer incremental value beyond established diagnostic approaches.

This review presents a structured framework for interpreting tongue-derived biological signals within the oral-systemic axis and outlines the methodological standards necessary for responsible clinical translation (Table 5). Clinical applicability should be considered only after validation through well-designed longitudinal cohort studies, standardised sampling protocols, phenotyping procedures and independent external replication.

**Table 5:** Minimum methodological requirements for future tongue-derived biomarker studies.

Domain	Minimum requirement	Rationale / evidence from this review
Study design	Longitudinal with ≥2 time points (baseline + follow-up)	All 25 included studies are cross-sectional; no data on within-individual temporal dynamics
Tongue sampling protocol	Standardised: fasting/morning, specific anatomical location (e.g., middle dorsum), fixed number of strokes (e.g., 5-10)	Sampling methods varied widely across studies (swabs, scrapers, toothbrushes; 1-30 strokes), limiting comparability
Confounder adjustment	PPI/antibiotic use (excluded if <3 months OR adjusted); oral hygiene (frequency/method); diet (validated questionnaire); smoking; alcohol	Consistently inadequate across all studies; see "Missing Confounders" in Table 1, Table 2
Phenotype classification	Standardised system (colour: white/yellow/other; thickness: thin/thick; moisture: moist/dry/greasy)	Phenotype modifies microbial composition^11^^,^^20^^,^^24^^,^^27^; current systems are incomparable
External validation	Independent cohort from different geographic/ethnic population	None of 25 studies performed external validation; 96% East Asian only
Sample size justification	A priori power calculation for primary microbiome outcome (accounting for multiple comparisons)	Current sample sizes range 14-1199 with no statistical justification
Oral health status	Periodontal examination (e.g., BPE/PISA) + caries count + denture/partial status	Periodontal disease excluded in only 2/25 studies; major potential confounder
Sequencing standards	Minimum depth specified; negative controls (extraction blanks, no-template controls); positive controls	Not reported in most studies; essential for reproducibility

Minimum methodological requirements were derived from a critical synthesis of limitations identified across all 25 included studies. Domains were selected based on consistent inadequacies in the evidence base (see Table 1, Table 2, Table 3 and Supplementary Tables S2-S4 for detailed study-level appraisal). PPI, proton pump inhibitor. Parenthetical citations refer to studies in the main reference list demonstrating phenotype-dependent effects.

## Conclusions

Based on the available evidence, tongue-derived signatures are best interpreted within a phenotype-microbiome-metabolome framework, in which the tongue-coating phenotype represents the visible ecological substrate, microbial dysbiosis reflects community-level biological variation, and metabolomic or proteomic alterations capture functional host-microbial outputs.

Gastrointestinal disease status may affect this triad directly, through systemic inflammation and altered host physiology, and indirectly through confounding factors such as medication use, diet, oral hygiene and disease-related behavioural changes. This framework facilitates a structured interpretation of tongue-derived signals while preventing their overclassification as diagnostic biomarkers.

A principal finding of this review is that tongue-derived signatures represent phenotype-associated, cross-sectional associations rather than clinically validated biomarkers. Apparent differences across disease stages primarily result from comparisons between heterogeneous patient populations and remain vulnerable to confounding influences.

From a clinical perspective, tongue examination remains valuable for oral pathology screening. However, current evidence does not support the use of tongue coating characteristics or tongue-derived microbiome profiles for diagnosing gastrointestinal diseases. These signatures should be interpreted as exploratory indicators of oral-gut axis interactions rather than as clinically actionable diagnostic tools.

Future research should prioritise longitudinal intra-individual designs, standardised and quantitative phenotype metrics, rigorous adjustment for oral and systemic confounders, and independent external validation. These approaches are essential to establish the temporal dynamics, mechanistic relevance and potential clinical applicability of tongue-derived signals.

## Declaration of competing interest

None disclosed.

## Appendix Supplementary materials

Supplementary File Supplementary Text S1. Search Terms Strategy Figure S1. PRISMA-ScR Flow Diagram Illustrating the Study Selection Process. Table S1. PRISMA-ScR Checklist Table S2. Methodological Appraisal of Studies on Tongue-Derived Microbial Signatures in Gastrointestinal Malignancies Table S3 Methodological Appraisal of Studies on Tongue-Derived Microbial Signatures in Inflammatory and Precancerous Gastrointestinal Conditions Table S4 Methodological Appraisal of Multi-Omics Studies on Tongue-Derived Signatures in Gastrointestinal Disease Image, application 1

## Footnotes

Supplementary material associated with this article can be found in the online version at doi:10.1016/j.identj.2026.109762.
