# Supplementary material for: Human Lingual Biofilm Signatures in Gastrointestinal Disease: A Scoping Review
Source: Int Dent J. 2026 Jul 28;76(5):109762. doi: 10.1016/j.identj.2026.109762 (PMC13446325; doi:10.1016/j.identj.2026.109762)
Supplement: Supplementary file 1 [file mmc1.docx]

**Supplementary File S1**

**Human Lingual Biofilm Signatures in Gastrointestinal Disease: A Scoping Review**

**Supplementary Text S1: Search Terms Strategy**

**PubMed/MEDLINE Search Strategy**

(("Tongue"[Mesh] OR "Tongue Coating"[tiab] OR "tongue coating"[tiab]

OR "dorsal tongue"[tiab] OR "tongue microbiome"[tiab]

OR "tongue microbiota"[tiab])

AND

("Microbiota"[Mesh] OR "Microbiome"[Mesh]

OR microbiota[tiab] OR microbiome[tiab]

OR metagenomic*[tiab] OR metaproteomic*[tiab]

OR metabolomic*[tiab] OR lipidomic*[tiab]

OR proteomic*[tiab] OR transcriptomic*[tiab]

OR "functional pathway*"[tiab]

OR "network analysis"[tiab]

OR "multi-omic"[tiab]

OR "omics"[tiab])

AND

("Gastrointestinal Diseases"[Mesh]

OR "Gastrointestinal Neoplasms"[Mesh]

OR gastritis[tiab]

OR "intestinal metaplasia"[tiab]

OR dysplasia[tiab]

OR "precancerous lesion*"[tiab]

OR "precancerous condition*"[tiab]

OR adenoma[tiab]

OR carcinoma[tiab]

OR "gastric cancer"[tiab]

OR "colorectal cancer"[tiab]

OR "esophageal cancer"[tiab]

OR "digestive system tumor*"[tiab]))

Filters:

• Publication date from 2010/01/01 to 2025/12/31

• Humans

• English language

**Scopus Search Strategy**

TITLE-ABS-KEY (

("tongue coating" OR "dorsal tongue" OR "tongue microbiome" OR "tongue microbiota")

AND

(microbiota OR microbiome OR metagenomic* OR metabolomic*

OR lipidomic* OR proteomic* OR transcriptomic*

OR "functional pathway*" OR "network analysis" OR "multi-omic" OR omics)

AND

(gastritis OR "intestinal metaplasia" OR dysplasia

OR "precancerous lesion*" OR adenoma

OR carcinoma OR "gastric cancer"

OR "colorectal cancer" OR "esophageal cancer"

OR "digestive system tumor*")

)

Limits:

• Year: 2010–2025

• English

• Article

**Embase Search Strategy**

('tongue'/exp OR 'tongue coating':ti,ab OR 'dorsal tongue':ti,ab

OR 'tongue microbiome':ti,ab OR 'tongue microbiota':ti,ab)

AND

('microbiome'/exp OR 'microbiota'/exp

OR metagenomic*:ti,ab OR metabolomic*:ti,ab

OR lipidomic*:ti,ab OR proteomic*:ti,ab

OR transcriptomic*:ti,ab OR 'multi-omic':ti,ab

OR 'functional pathway*':ti,ab OR 'network analysis':ti,ab)

AND

('gastrointestinal disease'/exp OR 'gastrointestinal cancer'/exp

OR gastritis:ti,ab OR 'intestinal metaplasia':ti,ab

OR dysplasia:ti,ab OR adenoma:ti,ab

OR carcinoma:ti,ab OR 'gastric cancer':ti,ab

OR 'colorectal cancer':ti,ab OR 'esophageal cancer':ti,ab)

Limits:

• Humans

• English

• 2010–2025

**Web of Science Search Strategy**

TS=(

("tongue coating" OR "dorsal tongue" OR "tongue microbiome" OR "tongue microbiota")

AND

(microbiota OR microbiome OR metagenomic* OR metabolomic*

OR lipidomic* OR proteomic* OR transcriptomic*

OR "functional pathway*" OR "network analysis" OR "multi-omic" OR omics)

AND

(gastritis OR "intestinal metaplasia" OR dysplasia

OR "precancerous lesion*" OR adenoma

OR carcinoma OR "gastric cancer"

OR "colorectal cancer" OR "esophageal cancer"

OR "digestive system tumor*")

)

Timespan: 2010–2025

Language: English

Document type: Article

**EBSCOhost (CINAHL, Academic Search)**

(tongue coating OR dorsal tongue OR tongue microbiome OR tongue microbiota)

AND

(microbiota OR microbiome OR metagenomic* OR metabolomic*

OR lipidomic* OR proteomic* OR transcriptomic*

OR "functional pathway*" OR "network analysis" OR "multi-omic")

AND

(gastritis OR intestinal metaplasia OR dysplasia

OR precancerous lesion* OR adenoma

OR carcinoma OR gastric cancer

OR colorectal cancer OR esophageal cancer)

Limiters:

• Published Date: 20100101–20251231

• English Language

• Peer-reviewed

• Human

**Identification of studies via databases and registers**

Records identified through database searching

PubMed (n = 168)

Scopus (n = 214)

Embase (n = 179)

Web of Science (n = 131)

EBSCOhost (n = 50)

January 2010 and December 2025

Total Records Identified: (n = 742)

Additional records identified via manual search (n = 6)

Total records (n = 748)

Duplicates removed (n = 221)

Records after duplicates removed (n = 527)

**Identification**

Records screened (n = 527)

Records excluded at title/abstract stage (n = 468)

**Screening**

*Full-text articles excluded (n = 34)*

*Reasons:*

No tongue sampling (n = 14)

Saliva/plaque only (n = 8)

Animal studies (n = 6)

Review/commentary (n = 3)

No molecular analysis (n = 3)

Full-text articles assessed (n = 59)

**Eligibility**

Studies included in scoping review (n = 25)

**Included**

**Figure S1. PRISMA-ScR flow diagram illustrating the study selection process.** Records were identified through database searching (PubMed, Scopus, Embase, EBSCOhost, and Web of Science) for studies published between 2010 and 2025. A final search update was performed in February 2026. After duplicate removal and title/abstract screening, full texts were assessed for eligibility based on predefined inclusion and exclusion criteria. Studies that met the eligibility criteria were included in the final scoping synthesis.

**Table S1** PRISMA-ScR Checklist

| **Item** | **PRISMA-ScR Requirement** | **Reported in Manuscript** |
| --- | --- | --- |
| Title | Identify the report as a scoping review | Title: Page 1 |
| Abstract | Structured summary | Abstract: Page 2 |
| Rationale | Describe rationale | Introduction; Pages 3-4 |
| Objectives | State objectives/questions | Pages 3-4 |
| Protocol and registration | Provide registration information | Methods 2.1; OSF Public DOI <https://doi.org/10.17605/OSF.IO/BW8QV> |
| Eligibility criteria | Specify inclusion/exclusion criteria | Methods 2.3; Page 4 |
| Information sources | Describe databases and dates | Methods 2.2; Page 4 |
| Search | Present full search strategy | Supplementary File S1 |
| Selection of sources | Describe the screening process | Methods 2.4; Page 4 |
| Data charting process | Describe extraction/charting | Methods 2.4; Page 4 |
| Data items | List variables extracted | Methods 2.4; Tables 1-3; 4; Tables on pp. 21-27 |
| Critical appraisal | Describe appraisal approach | Methods 2.4; Tables S1-S3; Page 4; Supplementary Tables S1-S3 |
| Synthesis | Describe synthesis methods | Results and Discussion; Pages 5-14 |
| Selection of sources results | Report screening numbers | Figure S1+ Results; Page 5; Figure S1 (Supplementary) |
| Characteristics of sources | Present characteristics | Tables 1-3; Pages 21-27 |
| Critical appraisal results | Present appraisal findings | Supplementary Tables S1-S3 |
| Results of individual sources | Summarize findings | Results Section, pages 5-8 |
| Synthesis of results | Summarize themes/patterns | Results Sections 3.1-3.5; Pages 6-8 |
| Summary of evidence | Summarize key findings | Discussion: Pages 8-14 |
| Limitations | Discuss limitations | Discussion 4.4; Pages 11-13 |
| Conclusions | Interpret results and implications | Conclusions: Page 14 |
| Funding | Describe funding sources | Funding statement page 15 |

**Table S2** Methodological Appraisal of Studies on Tongue-Derived Microbial Signatures in Gastrointestinal Malignancies

| **Study** | **Inclusion criteria** | **Setting** | **Exposure validity** | **Outcome validity** | **Confounders identified** | **Confounders addressed** | **Statistical analysis** | **Overall concern** | **Key limitations** |
| --- | --- | --- | --- | --- | --- | --- | --- | --- | --- |
| **Liu F. et al. (2025)** | Yes | Yes | Yes | Yes | Yes | Partial | Yes | Moderate | No PPI/oral hygiene adjustment; cross-sectional design |
| **Ma Y. et al. (2025)** | Yes | Yes | Yes | Yes | Yes | Partial (PSM) | Yes | Moderate | No PPI/oral hygiene; no external validation |
| **Wang H. et al. (2025)** | Yes | Yes | Yes | Yes | Yes | Partial | Yes | Moderate | Missing BMI/oral hygiene adjustment; small sample |
| **Chen Q. et al. (2024)** | Yes | Yes | Yes | Yes | Yes | Partial | Yes | Moderate | No PPI/oral hygiene; no external validation |
| **Nie F. et al. (2022)** | Yes | Yes | Yes | Yes | Yes | No | Yes | High | No control group; confounders not adjusted |
| **Zhang Y. et al. (2022)** | Yes | Yes | Yes | Yes | Yes | Partial | Yes | Moderate | Missing PPI/oral hygiene adjustment |
| **Chung M. et al. (2021)** | Yes | Yes | Yes | Yes | Yes | Partial | Yes | Moderate | No PPI/oral hygiene; within-subject design limitations |
| **Xu S. et al. (2021)** | Yes | Yes | Yes | Yes | Yes | Partial (PSM) | Yes | Moderate | No PPI/oral hygiene; no external validation |
| **Lu H. et al. (2019)** | Yes | Yes | Yes | Yes | Yes | Partial | Yes | Moderate–High | Small sample; missing confounders |
| **Hu J. et al. (2015)** | Yes | Yes | Yes | Yes | Yes | Partial | Yes | Moderate–High | Limited confounder control; subset sequencing |
| **Han S. et al. (2014)** | Yes | Partial | Yes | Yes | No | No | Partial | High | Very small sample; no confounder adjustment |

**Table S3** Methodological Appraisal of Studies on Tongue-Derived Microbial Signatures in Inflammatory and Precancerous Gastrointestinal Conditions

| **Study** | **Inclusion criteria** | **Setting** | **Exposure validity** | **Outcome validity** | **Confounders identified** | **Confounders addressed** | **Statistical analysis** | **Overall concern** | **Key limitations** |
| --- | --- | --- | --- | --- | --- | --- | --- | --- | --- |
| Yanan W. et al. (2023) | Yes | Yes | Yes | Yes | Yes | No | Yes | Moderate–High | No confounder adjustment |
| Xiao X. et al. (2022) | Yes | Yes | Yes | Yes | Yes | Partial | Yes | Moderate | Missing PPI/oral hygiene |
| Xiao P. et al. (2022) | Yes | Yes | Yes | Yes | Yes | Partial | Yes | Moderate | Confounders excluded but not adjusted |
| Kang et al. (2022) | Yes | Yes | Yes | Yes | Yes | Partial | Yes | Moderate | Missing PPI/oral hygiene |
| Hao et al. (2021) | Yes | Yes | Yes | Yes | Yes | Partial | Yes | Moderate | No validation; missing confounders |
| Cui et al. (2019) | Yes | Yes | Yes | Yes | Yes | Partial | Yes | Moderate–High | Limited confounder adjustment |

**Table S4** Methodological Appraisal of Multi-Omics Studies on Tongue-Derived Signatures in Gastrointestinal Disease

| **Study** | **Inclusion criteria** | **Setting** | **Exposure validity** | **Outcome validity** | **Confounders identified** | **Confounders addressed** | **Statistical analysis** | **Overall concern** | **Key limitations** |
| --- | --- | --- | --- | --- | --- | --- | --- | --- | --- |
| You Z. et al. (2024) | Yes | Yes | Yes | Yes | Yes | No | Yes | Moderate–High | No PPI/oral hygiene; no validation |
| Chen Q. et al. (2024) | Yes | Yes | Yes | Yes | Yes | Partial | Yes | Moderate | No PPI/oral hygiene |
| Hao Y. et al. (2021) | Yes | Yes | Yes | Yes | Yes | Partial | Yes | Moderate | No validation; confounders missing |
| Jain V. et al. (2023) | Yes | Yes | Yes | Yes | Yes | Partial | Yes | Moderate | Tissue vs coating heterogeneity |
| Chen J. et al. (2024) | Yes | Yes | Yes | Yes | Yes | Partial | Yes | Moderate | Limited confounder adjustment |
